# Soliciting Diaries for “Real-Time” Insights Into the COVID-19 Pandemic: Methodological Reflections on Using Digital Technologies to Engage the Public

**DOI:** 10.3389/ijph.2024.1606912

**Published:** 2024-09-25

**Authors:** Andrea Kaiser-Grolimund, Bettina Schwind, Laura Kämpfen, Anna Lea Morgan, Giovanni Spitale, Nikola Biller-Andorno, Sonja Merten

**Affiliations:** ^1^ Swiss Tropical and Public Health Institute (Swiss TPH), Basel, Switzerland; ^2^ Department of Public Health, University of Basel, Basel, Switzerland; ^3^ Department of Social Sciences, University of Basel, Basel, Switzerland; ^4^ Institute of Biomedical Ethics and History of Medicine, University of Zurich, Zurich, Switzerland

**Keywords:** qualitative solicited diaries, digital technologies, public perception, Switzerland, COVID-19, alternative narratives

## Abstract

**Objectives:**

To discuss the opportunities and challenges of the qualitative solicited diary approach using digital technologies as an innovative way to access insights into people’s lives and their unique stories in times of COVID-19-related social distancing in Switzerland.

**Methods:**

This case study provides reflective arguments from a social science perspective for the selection of different (digital) diary designs to optimize data collection in the SNSF-funded project “PubliCo - an experimental online platform for COVID-19-related public perception.”

**Results:**

The findings highlight some opportunities of using (digital) diaries, such as empowering participants, gathering real-time data, and ethical and methodological challenges when it comes to gaining access to alternative narratives.

**Conclusion:**

To gain in-depth insights during a crisis and to reach the lesser-heard voices that are central to democratic debates, it is necessary to adapt data gathering methods and build trust with diverse communities. While digital technologies open up new possibilities for public engagement, there is a need to think critically how data gathering is approached, how trustworthy the results are, and whose voices are captured, amplified, or left out.

## Introduction

Long before COVID-19, social scientists researching epidemics have been grappling with the methodological, epistemological, theoretical, and ethical issues that arise in the study of such crises [1]. One of the central questions has been how to best capture the heterogeneity of experiences and perspectives during a health crisis.

Every epidemic has an official outbreak narrative that according to Wald, “begins with the identification of an emerging infection, includes discussion of the global networks throughout which it travels, and chronicles the epidemiological work that ends with its containment” ([2], p.2). While this narrative relates to the design of the response pathways and their justification, it is intertwined with power and inequalities when it comes to who gets to determine the narrative [3]. Similarly, the metaphor of an infodemic can be understood as a powerful form of a pandemic narrative that simplifies various aspects of social behavior in a complex crisis, equating basic assumptions of viral spread with the circulation of miss/disinformation (cf., [4]), often loudly amplified through digital media. In contrast, our objective is to examine the intricacies of “alternative narratives” (cf., [3]) of “multiple publics” in Switzerland (for a critical discussion on publics and public sphere, see [5]). Compared to the official outbreak narrative which insists on a single explanatory plot line, we are interested in exploring the variety of narratives that pop-up throughout the pandemic course [6]. These narratives belong to “pandemic publics” imagined throughout the course of the COVID-19 pandemic in Switzerland (for a discussion on pandemic publics in Kenya, see [7]), as such they challenge simplistic notions of public engagement (cf., [8]). But how can we access these pluralities of voices during contact restrictions in real-time? And what kind of qualitative data can we gather?

This paper critically examines the opportunities and challenges of the qualitative solicited diary method using digital technologies in the context of COVID-19, focusing on whether it can ensure participation of “publics” in research and if it can accommodate democratic ideals like freedom of speech and equality in emergency preparedness and management. The discussions are based on our experience as health social scientists using the solicited diary method in the interdisciplinary “PubliCo” project, exploring different (digital) approaches.

### Potentials of Qualitative Solicited Diaries in Participatory Health Research

The diary method is a versatile tool for qualitative and quantitative social and health research [9]. Described as “a flexible tool for collecting rich data” ([10], p.2), it can vary in its purpose and can be either open/unsolicited or solicited [11]. While historians are interested in unsolicited diaries that serve as a “time capsule, revealing the lived experience of an historical epoch, such as […] the heartrending diary of the young captive Anne Frank” ([9], p.21), social and health scientists most often use “solicited” or “commissioned” diary methods to obtain more precise data focused on specific topics [9]. The length of solicited diaries can range from 1 day to several months. To gain insights into people’s thoughts and experiences of everyday life, diaries in qualitative health research may be adapted in terms of the techniques used to gather the data (cf., [11]). This may include the use of online and web-based technologies, as well as social sharing sites or blogs [10]. Although blogs differ in their performative act from the intimate, private, and unsolicited diaries - like those of Anne Frank, Zlata Filipović, or Frida Kahlo -, blogging “has re-energized and popularized the traditional form of diary keeping and might be regarded as the twenty-first-century equivalent of an unsolicited diary” ([10], p.61). Web-based technologies can quickly gather data and reach out to new diarists, they are cost-effective, user-friendly, and efficient in reaching large audiences (cf., [10–12]). Digital forms of diary collection, including mobile visual and audio journaling, can ensure critical, emancipatory and democratic ideals in the research process (cf., [13]).

Depending on their pre-defined structure and the way they are analyzed, qualitative diaries may provide us with a “deeper understanding of a person’s actions, experiences, thoughts, and feelings around a specific topic over time” ([14], p.1449). As such their insights essentially differ from the material gathered through in-depth interviewing at a specific moment of time (cf., [15]). During the COVID-19 pandemic, research projects explored this approach from different disciplinary perspectives (e.g., [16–21]), highlighting its potential to shed light on understandings of “authorities, institutions, discourses, standards, and codes” during a crisis ([17], p.397).

## Methods

The PubliCo project, based on an experimental online platform launched during the COVID-19 pandemic in Switzerland, aimed to collect data on “public perception” and to provide a bidirectional flow of information, by giving feedback and information after participation in a survey and allowing the aggregated data to be exposed on the platform, and by publishing findings in policy briefings on its open platform. The project sought to contribute to the fight against the infodemic in Switzerland [22]. It adopted an exploratory, transdisciplinary, multi-stakeholder approach, collecting real-time data through online survey modules, solicited diaries, and reflections based on open-ended prompts. The research team included ethicists, public health experts, philosophers, medical doctors, sociologists and anthropologists with their perspectives being rooted in different epistemologies.

The exploratory approach allowed the research team to test different formats of the diary method to adapt to people’s shifting needs and motivations over the course of the pandemic. Our aim was not to achieve a representative sample, but to explore in-depth how narratives could be captured. In a first phase, the project explored the diary approach during the spring and summer of 2020. Three social anthropology and urban studies students, some of them co-authoring this paper, supported the project by writing diaries themselves, critically exploring different ways of writing against the background of the existing literature on the approach. Two additional diary authors were recruited through personal contacts.

The first version of the PubliCo online platform launched in late 2020, and was followed by a second diary phase that lasted until summer 2021. Eleven diarists were recruited using different entry points such as associations, cantonally funded offices and personal contacts, followed by a snowball sampling. The diarists wrote or recorded weekly entries for 4 weeks. Their participation was accompanied by a personal exchange with the project team via phone or email.

Participants were encouraged to either upload their entries electronically to the online platform (https://publico.community/), submit texts on paper or via email, or share their audio recordings via mobile phone. In addition to an informed consent form, participants were provided with a brief guide that asked them to record their daily concerns, emotions and experiences on a weekly basis.

Midway through the project, the research team revised the diary approach based on the experience gained. In this third diary phase more diaries were gathered, reducing the requirement to two entries over 2 weeks. As it became increasingly clear that the diarists needed a personal counterpart to write to, the project website was enhanced with a dedicated diary presentation, available in English, French and German, introducing researchers in a personal way with photos.

The research team explored strategies to access people’s pandemic perceptions by collecting one-off reflections through the anonymous PubliCo platform, some of which where solicitated through Facebook advertisements. In a short time, over 1,000 posts were received with people sharing their opinions on specific (prompted) topics. We had the opportunity to work directly with schools to recruit teachers who contributed one-off reflections on their daily struggles. Solicited face-to-face encounters between researchers and interested participants resulted in 10 additional “reflections” that were submitted once, including nine audio-reflections of participants experiencing language barriers and a handwritten account of an elderly man.

All narratives were pseudonymized and analyzed following thematic analysis [23], using MAXQDA. Data was initially coded by one researcher and discussed and complemented by the interdisciplinary study team.

## Results

### Diarists’ Characteristics

A total of 21 diarists were successfully enrolled, submitting entries over 2–4 weeks, with a minimum of one entry per week. More diary entries were submitted, but did not meet the minimum requirement of entries per week/over time. Participants who submitted at least two entries over time ranged in age from 16 to 85, with the majority (6 women, 1 man) between the ages of 66 and 75. In total, 16 women and 5 men participated. The majority of participants submitted entries via email or postal mail, one via audio and three via the PubliCo platform.

In this paper, we do not consider the one-off reflections and Facebook posts mentioned above as “diaries,” but they do help us in our methodological reflections.

### Expected Opportunities of Pandemic Diaries and Its Reality Check

Our analysis of diary entries revealed self-reflective accounts of negotiated daily dilemmas with established government rules that were consciously adapted to real life. Some entries entailed messages directed to those managing the pandemic and provided insights into the personal meaning-making processes:

I had the following thoughts about the whole “problem of the older people” […]. It is always emphasized that the OLD people should stay at home as a risk group, and again the OLD people and again the OLD people. This led to discrimination (e.g., mobbing or at least angry looks) against the older people by some (many?) as soon as they showed themselves in public, e.g., when shopping. In my opinion, the tenor should be: you old people, go for a walk so that you have fresh air and exercise, but observe the hygiene regulations and keep your distance. Then resentment of the young population would stop…

Hans-Peter, 76-85, May 2020 (Original quote in German, see Supplementary File S1)

**FIGURE 1:**
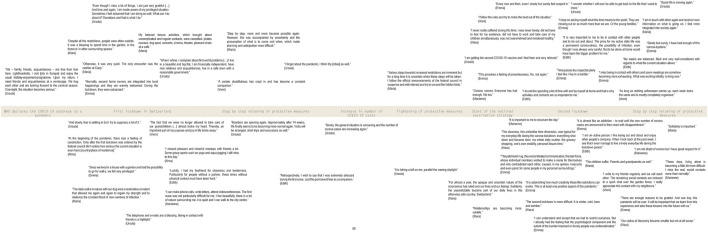
Timeline of diary entries by older women (Switzerland, 2021) (Supplementary Material S2 contains the original quotes in German) [28].

In addition, diary approaches allow access to “the social at a distance,” while the researcher is absent ([24], p.139). In a situation of “social distancing,” it can allow for a continuous collection of in-depth insights into the lifeworld, enabling “social listening” (cf., [25, 26]). The latter may inform communication strategies that better reflect the needs of specific “publics” and contribute to crisis management [25]. For example, qualitative diary studies during the pandemic may reveal which public health measures at what point in time cause emotional distress or an increase in social inequalities for particular groups. Such real-time and time specific recording is critical in the rapidly changing context of a pandemic [14, 27]. With a focus on the diary entries of older women, Figure 1 depicts a timeline that captures their shifting priorities and emotional distress over the course of the pandemic [28].

In our study, the diary approach was useful to better understand the changing complexities of the COVID-19 vaccination campaign and how its management affected older people’s emotions, decisions, and practices over time. In Switzerland, the national vaccination strategy prioritized people at risk for COVID-19 infections (people over 75 years of age or with pre-defined health conditions), with each canton adapting its vaccination strategy to its priorities. This led to different criteria in different cantons until June 2021. The older diary authors, living in different German speaking cantons of Switzerland and mostly in relatively stable financial situations, experienced the uncertain situation differently, expressing their frustrations, hope, and changes in attitudes over time as vaccines became more accessible. Urs’ entry reveals some underlying critiques and frustrations with the vaccination campaign:

My understanding of the drastic measures is also waning, especially since the infection figures have been falling for some time and vaccinations have now begun. Now, all of a sudden, I am no longer one of the particularly vulnerable people, because apparently the vaccine is still too scarce. I would like to be vaccinated, but at 66 I am too young.

Urs, 66–75, February 2021 (see Supplementary Material S1)

In contrast, Vreni expresses hope for a quick vaccination and reduced anxiety in daily activities: “Other than that, I hope every day that I stay healthy and the day comes when I get to go get vaccinated.” (Vreni 66–75, March 2021, see Supplementary Material S1) At the same time, Gertrud (76–85), who was eligible for vaccination at the very beginning, viewed the vaccination as a liberating gift that could bring a freer new year 2021. Being able to capture these frustrations and hopes in “real” time provided crucial insights into the diversity of impacts of vaccination policies on the “older public.”

Moreover, the diary method may offer access to tacit knowledge, which refers to aspects that participants may take for granted [27]. Understanding tacit knowledge is crucial for public health initiatives, because people in a crisis situation draw on past experiences shaped by local circumstances, which need to be considered alongside the authority of evidence in order to build resilient, people-centered health systems (cf., [29]). This implicit knowledge can be tapped when diarists describe everyday activities, as the thoughts of Erika on how to best get around do:

I took the train!!! With a mask and a lot of caution, but with the feeling of being a bit freer and more mobile again. My compromise was good. By bike to the station, so that I didn’t have to take the tram AND the bus.

Erika, 66–75, May 2020 (see Supplementary Material S1)

### Methodological and Epistemological Challenges

During the lockdown when we were exposed to drastic changes, moral dilemmas, and emotional vulnerability, it appeared that it was easier for participants to adhere to a writing schedule and to document events, catalyzing a process of self-reflection that was documented in the diary entries. As the crisis progressed and a “new normal” of routines derived (cf., [30]), diarists appeared to feel that their everyday lives no longer seemed special or exciting to report. This chronicisation of the crisis may be considered an important finding in itself, as Vreni’s entry reveals: “Unless something unforeseen comes up, every week resembles the next … ” (Vreni, 66–75, March 2021, see Supplementary Material S1). Some people became fed up with the pandemic and the diary approach ran the risk of not collecting enough data due to potential drop outs or peoples’ unwillingness to enroll (e.g., [31]). To address this, the team modified and shortened the diary format from 4 to 2 weeks, weighting the shortened engagement against tracking changes over time.

Although flexible and open, diary keeping may present difficulties to people who are used to other media. Even though today’s technologies are increasingly able to adapt to different groups of actors, they may not be accessible or appealing to all equally [27]. In our study for example, we had less difficulties in accessing older diarists. Yet, the approach risks lacking the voice of particularly vulnerable groups (cf., [12]). It is crucial to invest considerable time and effort in recruiting diverse voices, creating an “inclusive diary keeping design,” adapted to the particular needs of a target group.

The PubliCo project aimed to address these challenges by making participation options open which made the process more complex to participants. Therefore, while the project explored an inclusive approach to engage different groups such as through a migrant specific TV channel, it hindered equal participation due to its openness and complexity. Nevertheless, this openness should be critically considered when analyzing entries, as people wrote in different styles, from bullet point reports to extensive emotional elaborations.

To overcome these difficulties, the exploratory design allowed a second strategy in which people were asked to contribute reflections in a single entry or audio recording whereby we consciously accepted of losing the diary approach’s strength to capture changes over time. This allowed us to audio record the experiences of people whom the research team met in an internet café for people experiencing (or at risk of experiencing) poverty, when the social distancing rules were lifted. This personal approach helped building trust through personal relationships, while supporting the credibility of findings (cf., [32]).

Large numbers of people were reached via Facebook advertisements, however due to its inbuilt algorithms, this recruitment strategy may have led to the selection of participants who were more critical of containment measures - attracted to anonymity and short engagement. Their responses were brief, e.g., “nothing” or “none,” making it difficult to qualitatively analyze data or assess their perspective. It stripped us as social scientists of the central tools to assure trustworthiness of data, which is an empirical, contextual, and immersed perspective that allows for dense data gathering (cf., [33]).

Manually analyzing diary data in a short time frame requires a team of researchers or the development of NLP/AI-based solutions (cf., [26]), which necessitates an awareness of the strengths and limitations of both approaches or their combination (cf., [34]). Furthermore, if we seek to understand accounts over a period of time, we need to analyze the process of knowledge production inherent in the diary method. As with ethnographic research, we need to critically interrogate aspects of positionality, asking how the underlying power relationship between researchers and participants affects participants’ writing, an aspect that is not always explicitly visible in the diary entries [35]. Similarly, the research team’s perceptions of whose voices are considered “alternative” or “marginalized” need to be carefully reflected – being shaped by the same public discourse as the research participants while these perceptions may not necessarily be shared by the participants themselves.

### Critical Reflections on Trust and Public Participation

In the Swiss pandemic context, the discussion about data protection took on a new importance, and people’s concerns about their private information came up in PubliCo, as a short quote from a public comment after an online newspaper article about the project reveals: “Never trust the government and websites like this.” (Original quote in German, see [36]).

The project aimed to protect participants’ anonymity and promote transparency by collecting data through a secure online platform. This worked well for the quantitative part of the study, as people filled out an online questionnaire without digital fingerprinting technologies or for the one-off reflections with a more simple design. However, when it came to uploading personal diary entries, many participants seemed to prefer personal contact with the project team. This allowed them to clarify any uncertainties. The trust built up motivated diarists and assured their personal data would not be disclosed or misused. Additionally, many appreciated having someone to write for, and “being there” as a researcher may have brought more value than material tokens of gratitude during a pandemic crisis (cf., [37]). As it became possible to meet again physically, this opened up new possibilities for recruitment. However, while the scalability and the relatively low costs of the online diary method could be cited as an added benefit, as soon as the recruitment strategies became more personal and time-intensive, they became more costly.

When recruiting diarists and analyzing their entries, it is crucial to consider the potential psycho-social side effects of making them write about their experiences. Many of the older diarists thanked the research team noting that participating was a good experience for them. The self-reflexive approach of the diary-method may have an interventional aspect; diaries have the potential to provide participants a “space to develop insight into their lives and give voice to experience” ([35], p.675, refering to [38]). Such interventions are the result of intimate confessions, as diaries “are the space to say what cannot be said out loud” ([35], p.675).

Thereby, the diary method can help scientists in understanding notions of public health interventions, while it may aid participants to find their role and their path in such an extreme situation. At the same time we as social researchers must be cautious about whom we address as “the public,” and be aware that collecting their voices is not enough to ensure an equal engagement in crisis management.

## Discussion

This case study critically reflected on the opportunities and challenges of using qualitative solicited diaries as a (digital) method for future pandemic preparedness to support democratic ideas of debate and crisis management. The focus was on the lessons learnt and not on the analysis of the diary content. Digital diaries can foster trust in science and governments in times of crisis, as they allow for the free expression of diverse pandemic publics about the process and context of a public health emergency (cf., [39]).

We believe that if diarists can be successfully recruited, this approach is in line with the UN Sendai Framework for disaster and risk reduction 2015–2030 [40], which emphasizes the importance of people-centred multi-hazard communication mechanisms and social technologies that are developed through a participatory process. Digital diaries can provide an online space for diverse understandings of “good” citizenships, highlighting the contextuality of democratic debate and relationality of politics (cf., [17]). However, to foster digital democratic governance, it is critical to reflect how platforms as PubliCo are situated and applied within diverse on- and offline publics to live up to democratic ideals of equal participation, specifically during a crisis.

If designed well, the use of such digital technologies can be cost effective and fast. Yet, there remains the question of how far the digital approach of the PubliCo project, specifically the reflections gathering via Facebook, truly promotes equal participation, as media companies’ algorithmic governance influence the digital space, citizens choices, and political opinions (cf., [41]). Moreover, a possible self-selection bias could be mitigated by diversifying recruitment strategies. Social scientists face challenges in identifying research boundaries and assessing which voices are amplified and/or silenced (cf., [42]). Recruitment for diaries needs to be adapted to specific social groups and contexts, as their access to digital methods and trust in institutions can vary. This is in line with Chambers and colleagues, who used digital diaries during the COVID-19 pandemic and summarized that the participation of certain groups required personal relationships, time, and adaptation [43]. If tools and approaches are not flexible, data gathering may conflict with democratic ideas of equality and lead to certain groups being unheard. Additionally, researchers should be cautious about drawing conclusions for crisis management in the name of “the public,” as it is in itself a dynamic and fluid concept across time, space and context [44].

Our findings show that the “new normal” changed the motivation to keep a diary and that trust (worthiness) is a complex crucial factor for data gathering during a public health crisis. Access to quieter narratives through diaries requires a certain proximity and trust, which is essential for qualitative social science research to provide an emic perspective and explain the demands of diary keeping.

### Conclusion

We conclude that the diary method, using digital technologies, can provide valuable and timely insights into the lives of diarists during an ongoing crisis. It can help to gather thorough feedback on public perceptions. However, it requires much time and resources for recruitment (and analysis). Trust may help motivate people to continue writing. It shows that certain insights can only be gained by investing in personal relationships, which is challenging in crisis situations – but not impossible. This case study has explored the use of solicited diaries through various non/digital means for future crisis preparedness and management, without claiming that the method can address all shortcomings. The use of digital technologies provides an opportunity to invite large numbers of people to contribute to public debates. However, low cost outreach neither guarantees “good data” nor that everyone will be heard equally. These considerations and the resulting mitigation strategies need to be part of a well-considered, planned, and executed recruitment strategy that can make or break the game of equal participation in debating crisis management in the future.
