# Supplementary material for: Soliciting Diaries for “Real-Time” Insights Into the COVID-19 Pandemic: Methodological Reflections on Using Digital Technologies to Engage the Public
Source: Int J Public Health. 2024 Sep 25;69:1606912. doi: 10.3389/ijph.2024.1606912 (PMC11484068; doi:10.3389/ijph.2024.1606912)
Supplement: Supplementary file 1 [file DataSheet1.zip › Supplementary Material S1.pdf]

Supplement 1: Original Quotes in German

p. 5

‘Zur ganzen ‚Altenproblematik‘ habe ich mir folgende Gedanken gemacht […] Man betont immer, dass die ALTEN als eine Risikogruppe zuhause bleiben sollen, und nochmals die ALTEN und immer wieder die ALTEN. Das führte dazu, dass von einigen (vielen?) eine Diskriminierung (z.B. Anpöbelung oder zumindest böse Blicke) gegenüber den Alten erfolgte, sobald sich diese in der Öffentlichkeit, z.B. beim Einkaufen, zeigten. M.E. sollte der Tenor sein: ihr Alten, geht ruhig spazieren, damit ihr frische Luft und Bewegung habt, beachtet aber die Hygienevorschriften und das Abstandhalten. Dann würden Ressentiments der jungen Bevölkerung aufhören...’.

(Hans-Peter, 76-85, May 2020)

p.5

‘Das Verständnis für die einschneidenden Massnahmen ist auch bei mir am Schwinden, zumal die Infektionszahlen seit geraumer Zeit sinken und inzwischen mit den Impfungen begonnen wurde. Jetzt gehöre ich plötzlich nicht mehr zu den besonders vulnerablen Personen, weil offenbar der Impfstoff noch zu knapp ist. Gerne würde ich mich impfen lassen, mit 66 Jahren bin ich aber zu jung.’

(Urs, 66-75, February 2021)

p.5

‘Ansonsten hoffe ich jeden Tag, dass ich gesund bleibe und der Tag kommt, an dem ich zum Impfen gehen darf.’

(Vreni, 66-75, March 2021)

p. 5

‘Ich bin Zug gefahren!!! Mit Maske und sehr viel Vorsicht, aber mit dem Gefühl wieder etwas freier und mobiler zu sein. Mein Kompromiss war gut. Mit dem Velo bis zum Bahnhof, dass ich nicht Tram UND Bus nehmen musste.’

(Erika, 66-75, May 2020)

p. 6

‘Solange nichts Unvorhergesehenes dazwischen kommt gleicht jede Woche der anderen…’

(Vreni, 66-75, March 2021)
