# Supplementary figures and images for: Soliciting Diaries for “Real-Time” Insights Into the COVID-19 Pandemic: Methodological Reflections on Using Digital Technologies to Engage the Public
Source: Int J Public Health. 2024 Sep 25;69:1606912. doi: 10.3389/ijph.2024.1606912 (PMC11484068; doi:10.3389/ijph.2024.1606912)

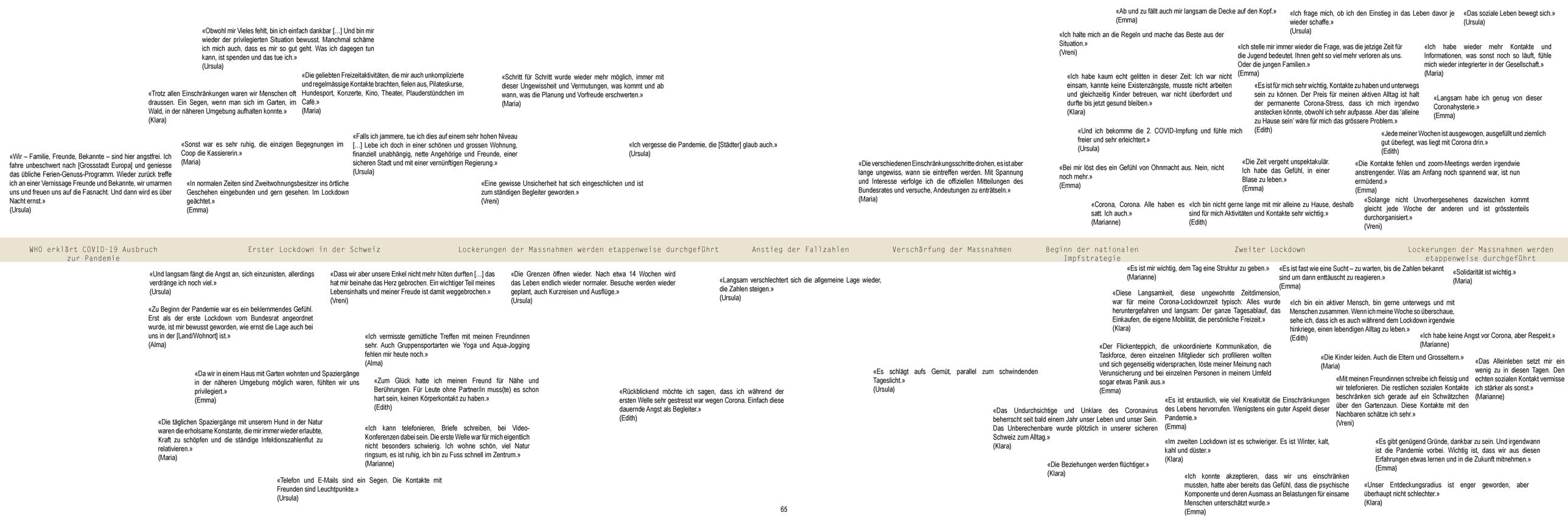

Supplement: Supplementary file 1 [file DataSheet1.zip › Supplementary Material S2.JPEG]
